# Neurofilament light chain as a mediator between *LRRK2* mutation and dementia in Parkinson’s disease

**DOI:** 10.1038/s41531-023-00572-3

**Published:** 2023-09-12

**Authors:** Dehao Yang, Haobo Xie, Sheng Wu, Chenxin Ying, Yiqun Chen, Yaoying Ge, Ruotong Yao, Kun Li, Zihan Jiang, Guangyong Chen

**Affiliations:** 1 Department of Neurology, The Second Affiliated Hospital, Zhejiang University School of Medicine, Hangzhou, China; 2 The First School of Medicine, School of Information and Engineering, Wenzhou Medical University, Wenzhou, China; 3 The Second School of Medicine, Wenzhou Medical University, Wenzhou, China; 4 Department of Neurology, The Third Affiliated Hospital of Wenzhou Medical University, Wenzhou, China

**Keywords:** Mutation, Parkinson's disease, Databases, Cognitive ageing

## Abstract

Elevated neurofilament light chain (NfL) levels have been associated with dementia in idiopathic Parkinson’s disease (iPD). To examine the baseline and longitudinal changes in NfL levels in *GBA*-PD, *SNCA*-PD, and *LRRK2*-PD and further investigate the association between these genetic mutations, NfL, and dementia in PD. We analyzed data from the Parkinson’s Progression Markers Initiative (PPMI), including 184 healthy controls (HC) and 617 PD categorized as iPD (*n* = 381), *LRRK2*-PD (*n* = 142), *GBA*-PD (*n* = 76) and *SNCA*-PD (*n* = 18). Analysis of covariance (ANCOVA) or linear mixed-effect models were used to compare the baseline or dynamic NfL levels between groups. We then explored the relationship between genetic mutations, serum NfL levels, and conversion to dementia using mediation analysis. After adjusting for confounding factors, *SNCA*-PD exhibited higher baseline serum NfL levels than iPD. Regarding longitudinal changes, *SNCA*-PD showed the highest increase rate in estimated NfL levels (2.43 pg/mL per year), while *LRRK2*-PD experienced the slowest increase rate (0.52 pg/mL per year). Mediation analysis indicated that higher estimated NfL level changes were associated with faster cognitive decline (*β* = 0.591, *p* = 0.026). Specifically, the relationship between *LRRK2* and dementia was mediated by the estimated NfL level change (*β* = −0.717, *p* < 0.05). Longitudinal changes in serum NfL levels may serve as a biomarker for cognitive decline in Parkinson’s disease. Moreover, compared to iPD, the slower progression of dementia in *LRRK2*-PD may be partially attributed to a slower increase in NfL levels.

## Introduction

Cognitive decline is a prevalent non-motor impairment in Parkinson’s disease (PD), where patients may experience conversion from normal cognitive function to mild cognitive impairment (MCI) and eventually to Parkinson’s disease dementia (PDD) over time^1^. Current evidence suggests that with disease progression, the incidence of PDD tends to rise^2,3^. Tracking the progression of cognitive decline and assisting in clinical intervention requires a concise and accessible biomarker. Unfortunately, there are no reliable blood or cerebrospinal fluid (CSF) biomarkers for diagnosing and managing PDD in clinical practice.

Neurofilament light chain (NfL) has recently been proposed as a potential biomarker of neuroaxonal degeneration in multiple neurological disorders^4^. Specifically, in idiopathic Parkinson’s disease (iPD), several studies have demonstrated a correlation between blood NfL levels and motor and cognitive impairment^5–7^. Furthermore, baseline serum NfL levels and their longitudinal change have been identified as valuable biomarkers to predict the severity of cognitive decline and the likelihood of developing dementia. Higher NfL levels were associated with an increased risk of PDD^8,9^.

Recent research on mutations in *GBA*, *LRRK2*, and *SNCA* has elucidated specific pathways that shed light on the underlying mechanisms of PD. Among these mutations, *LRRK2* and *GBA* are the most common genetic risk factors for PD, while *SNCA* is relatively rare^10,11^. It has been observed that compared to iPD, PD with *LRRK2* pathogenic mutation (*LRRK2*-PD) tends to exhibit a milder phenotype, and PD with *GBA* pathogenic mutation (*GBA*-PD) and PD with *SNCA* pathogenic mutation (*SNCA*-PD) are more likely to develop severe cognitive impairment^12–14^. These mutations impact the progression of PD through various mechanisms, ultimately influencing the risk of developing PDD.

However, it remains unclear whether NfL plays a mediating role in the association between these mutations and cognitive decline. The primary objective of this study was to investigate whether PD with different mutations (*LRRK2*-PD, *GBA*-PD, or *SNCA*-PD) exhibit distinct serum NfL levels, both at baseline and during longitudinal change. Besides, we sought to investigate whether mutations have a mediating effect through NfL on conversion to dementia.

## Results

### Study participants

The demographic and clinical features of all participants are presented in Table 1. We compared the differences between healthy controls (HC, *n* = 184) and the overall PD group, as well as among the different PD subgroups, including iPD (*n* = 381), *GBA*-PD (*n* = 76), *LRRK2*-PD (*n* = 142), and *SNCA*-PD (*n* = 18) at baseline. Age, sex, and education years were comparable between the overall PD and HC groups. However, the overall PD group had significantly higher serum NfL levels (mean: 13.88 pg/ml, *p* = 0.001), higher Movement Disorders Society Unified Parkinson’s Disease Rating Scale (MDS-UPDRS) III scores (mean: 21.25, *p* < 0.001), and lower Montreal Cognitive Assessment (MoCA) scores (mean: 26.69, *p* < 0.001) compared to HC. Subgroup analysis among PD subgroups revealed that *SNCA*-PD were significantly younger (mean: 49.26 years) and had lower education levels (mean: 11.39 years). Male predominance was observed among subjects in the iPD (65.40%) and *GBA*-PD (56.60%) groups, while the *LRRK2*-PD group consisted predominantly of females (46.50%). PD with mutations had a longer disease duration than iPD (*p* < 0.001). Regarding serum NfL levels, the *LRRK2*-PD group had the highest value (mean: 15.25 pg/ml), while the *SNCA*-PD group had the lowest value (mean: 13.46 pg/ml), although the difference was not statistically significant (*p* = 0.113). Although the *SNCA*-PD group had the lowest MoCA score (mean: 23.78), there was no significant difference from other groups. The *LRRK2*-PD group had a lower MoCA score than the iPD group (*p* = 0.005). In addition, the *GBA*-PD (mean: 1.80) and *LRRK2*-PD (mean: 1.82) groups were associated with higher Hoehn and Yahr (H&Y) stages compared to the iPD group (mean: 1.56). The *GBA*-PD group also had higher MDS-UPDRS III scores (mean: 25.01) and MDS-UPDRS total scores (mean: 40.71) than the iPD group.

**Table 1:** Demographics and baseline characteristics between groups.

Variables	HC (*n* = 184)	Overall PD (*n* = 617)	iPD (*n* = 381)	*GBA*-PD (*n* = 76)	*LRRK2*-PD (*n* = 142)	*SNCA*-PD (*n* = 18)	*p* between HC and total PD	*p* among PD groups^A^
Age (years)	60.88 (11.08)	61.88 (9.97)	61.97 (9.66)^a^	61.63 (10.63)^a^	63.38 (9.32)^a^	49.26 (10.33)^b^	0.333	<0.001
Sex (male)	117 (63.60%)	367 (59.50%)	249 (65.40%)^a^	43 (56.60%)^a, b^	66 (46.50%)^b^	9 (50.00%)^a, b^	0.318	0.001
Education (years)	16.05 (2.91)	15.37 (3.56)	15.53 (2.90)^a^	16.14 (3.60)^a^	15.05 (4.60)^a^	11.39 (4.33)^b^	0.069	<0.001
Disease duration (Months)	NA	36.35 (41.61)	23.99 (24.09)^a^	48.73 (35.05)^b^	61.91 (64.43)^b^	47.89 (25.90)^b^	NA	<0.001
Serum NfL (pg/mL)	11.84 (6.58)	13.88 (8.41)	13.18 (7.19)	14.87 (10.60)	15.25 (9.66)	13.46 (9.92)	0.001	0.113
MoCA (baseline)	28.23 (1.10)	26.69 (2.86)	27.13 (2.34)^a^	26.33 (2.78)^a, b^	26.08 (3.16)^b^	23.78 (6.38)^a, b^	<0.001	0.002
H&Y stage	NA	1.66 (0.54)	1.56 (0.50)^a^	1.80 (0.54)^b^	1.82 (0.57)^b^	1.78 (0.73)^a, b^	NA	<0.001
MDS-UPDRS III score	1.18 (2.16)	21.25 (10.06)	20.85 (8.86)^a, b^	25.01 (12.24)^a^	20.11 (9.86)^b^	22.67 (19.14)^a, b^	<0.001	0.022
MDS-UPDRS total score	4.57 (4.43)	34.22 (15.33)	32.08 (13.25)^a^	40.71 (17.10)^b^	35.45 (15.92)^a, b^	42.50 (28.75)^a, b^	<0.001	0.001

Data are mean (SD) or *n* (%). Significance level for comparisons is *p* < 0.05.

*NfL* Neurofilament light chain, *MoCA* Montreal Cognitive Assessment, *H&Y* Hoehn and Yahr, *MDS-UPDRS* Movement Disorders Society Unified Parkinson’s Disease Rating Scale.

^A^Indicated continuous variables were compared using Kruskal–Wallis test, and categorical variables were compared with chi-square test. Multiple comparisons between four PD groups were adjusted by Bonferroni correction. Values with same letters are not statistically different (*p* > 0.008).

### Serum NfL at baseline between groups

After adjusting for age, sex, disease duration, and education, the *SNCA*-PD group (reference) had higher serum NfL at baseline than the iPD group (*β* = −6.471, *p* < 0.001). The differences between *SNCA*-PD and *GBA*-PD (*β* = −6.405, *p* = 0.032) and between *SNCA*-PD and *LRRK2*-PD (*β* = −5.715, *p* = 0.014) failed to reach significance after Bonferroni correction. No significant difference in serum NfL levels was observed between the other groups (Table 2, Supplementary Fig. 1).

**Table 2:** Comparisons of serum NfL at baseline using ANCOVA.

Reference group	group	β	Standard error	*p* value
*SNCA*-PD	iPD	−6.471	1.632	<0.001
	*GBA*-PD	−6.405	2.931	0.032
	*LRRK2*-PD	−5.715	2.301	0.014
*LRRK2*-PD	iPD	−1.188	0.709	0.094
	*GBA*-PD	0.546	1.221	0.655
*GBA*-PD	iPD	−1.336	0.897	0.137

ANCOVA was adjusted by age, sex, disease duration, and education.

### The relationship between serum NfL at baseline and different mutations in PDD

**Fig. 1:**
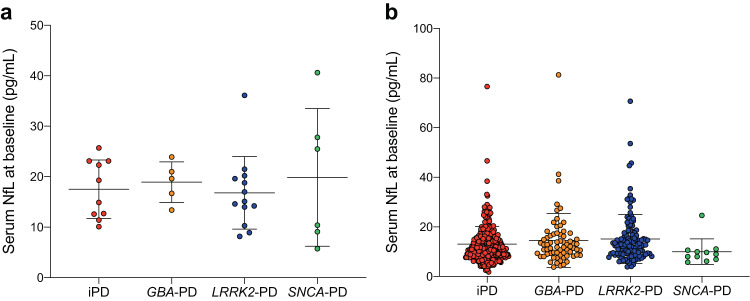
Comparisons of baseline serum NfL between iPD, *GBA*-PD, *LRRK2*-PD, and *SNCA*-PD. **a** Scatter plot for baseline serum NfL in PDD. **b** Scatter plot for baseline serum NfL in PD without dementia. Centre line denotes median, with whiskers for maximum and minimum.

We further explored whether different mutations were associated with different serum NfL levels at baseline in PDD. PD were divided into two subgroups based on the presence or absence of dementia at baseline. The results revealed no significant difference in serum NfL levels at baseline among the different mutations in PDD (Fig. 1a). Similarly, when comparing PD groups without dementia at baseline, no significant difference in serum NfL levels was observed (Fig. 1b).

### Estimated change of serum NfL

**Fig. 2:**
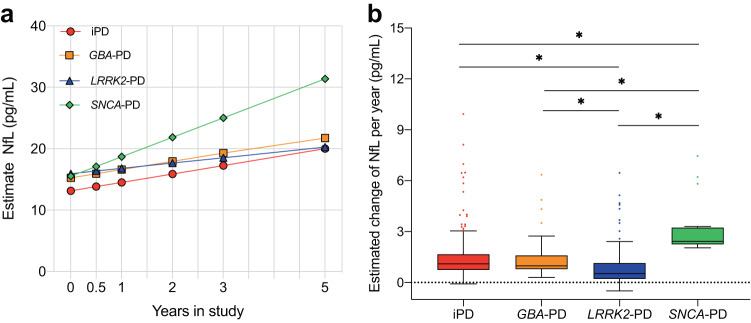
Comparisons of estimated change of serum NfL between iPD, *GBA*-PD, *LRRK2*-PD, and *SNCA*-PD. **a** Line graph for dynamic serum NfL. **b** Box plot for comparisons of estimated change of serum NfL. Centre line denotes median, with bounds of box for quartile and whiskers for the most extreme data point no more than 1.5x the interquartile range from the box.

We predicted the longitudinal profile of serum NfL using linear mixed-effect models (LMM) (Fig. 2). Mutations exerted a significant impact on the longitudinal change of NfL. The *SNCA*-PD group (2.43 pg/mL per year) showed the highest estimated change in NfL, while there was no difference in the longitudinal change of NfL between iPD (1.10 pg/mL per year) and *GBA*-PD (0.98 pg/mL per year) groups (*p* = 0.823). The lowest estimated NfL rate was observed in the *LRRK2*-PD group (0.52 pg/mL per year).

### Mediation analysis

**Fig. 3:**
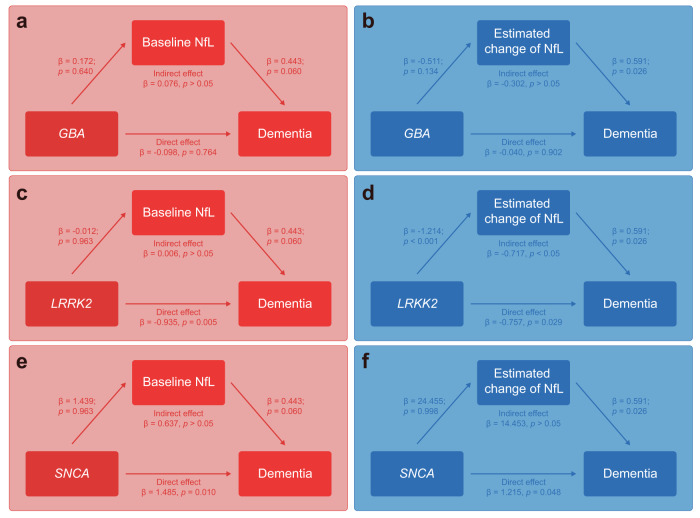
Mediation analysis of mutations for dementia through baseline serum NfL or estimated change of serum NfL. **a** GBA mutation for dementia through baseline serum NfL; **b** GBA mutation for dementia through estimated change of serum NfL; **c** LRRK2 mutation for dementia through baseline serum NfL; **d** LRRK2 mutation for dementia through estimated change of serum NfL; **e** SNCA mutation for dementia through baseline serum NfL; **f** SNCA mutation for dementia through estimated change of serum NfL.

The results of the mediation analysis are displayed in Fig. 3. For *GBA* mutation, no direct or indirect effect on dementia was found through either baseline NfL or estimated change of NfL (Fig. 3a, b). We observed that *LRRK2* mutation was directly associated with a lower risk of dementia (*β* = −0.757, *p* = 0.029), and its association with dementia was partly mediated (*β* = −0.717, *p* < 0.05) by the estimated change of NfL (Fig. 3d). However, baseline NfL did not have a significant mediation effect on the relationship between *LRRK2* mutation and dementia (Fig. 3c). *SNCA* mutation did not influence dementia via the mediation of either serum NfL or estimated change of serum NfL but exerted a direct effect (*β* = 1.215, *p* = 0.048) on dementia (Fig. 3e, f). Besides, in the pathway linking NfL to dementia, we observed that a higher estimated change in serum NfL levels was associated with an increased likelihood of conversion to dementia (*β* = 0.591, *p* = 0.026). However, we did not find a significant relationship between baseline NfL and conversion to dementia (*β* = 0.443, *p* = 0.060).

## Discussion

Building upon prior research findings, we aimed at analyzing whether there were differences in baseline or longitudinal changes in serum NfL levels among PD with different mutations and to determine if these differences impacted dementia. Initially, we compared the baseline serum NfL levels between different groups. The overall PD group had higher serum NfL levels than the HC group at baseline, consistent with a previous study^8,15^. Ma et al.^8^ reported that a higher serum NfL level was associated with a faster decline in MoCA score. Besides, recent studies indicated that *GBA*-PD and *SNCA*-PD experienced more severe cognitive impairment, whereas those with *LRRK2* mutation had milder symptoms^13,16^. Thus, we hypothesized that *GBA*-PD and *SNCA*-PD would have higher serum NfL levels while *LRRK2*-PD would have lower serum NfL levels at baseline. However, we did not observe significant differences in serum NfL levels between iPD, *GBA*-PD, *LRRK2*-PD, and *SNCA*-PD groups, possibly due to the differences in age and other demographic characteristics among the groups at baseline. Huang et al.^15^ confirmed that serum NfL levels were positively associated with age in both HC and PD, suggesting that higher serum NfL levels may be influenced by older age. We then used ANCOVA and adjusted for age, sex, education, and disease duration, to compare NfL levels between groups. It was found that *SNCA*-PD had higher serum NfL at baseline after adjustment compared to iPD, but the difference between the other groups did not reach statistical significance, consistent to some extent with our hypothesis. Furthermore, our study uncovered an additional finding regarding the variation in clinical scores among different mutations. Specifically, *GBA*-PD exhibited a more advanced H&Y stage and higher MDS-UPDRS III score and MDS-UPDRS total score, while *LRRK2*-PD exhibited lower MoCA score at baseline.

There is a rich literature available substantiating that PD have relatively higher serum NfL levels, and PDD is associated with even more elevated serum NfL levels compared to HC^8,9,15^. Therefore, we explored whether there were variations in serum NfL levels at baseline among PD with different mutations in PDD group. However, our analysis did not reveal any significant differences, which could be attributed to the limited sample size in one or more mutation groups.

There is an increasing consensus suggesting that baseline NfL levels represent a useful biomarker for assessing cognitive severity and prognosis in iPD^5,6,9^. Meanwhile, studies have shown that the temporal trajectory of NfL may be equally valuable in assessing cognitive decline, comparable to NfL levels at a single time point^8,17^. After comparing baseline serum NfL levels, we further explored whether there were differences in the longitudinal change of NfL among PD with different mutations. We utilized a LMM to evaluate the longitudinal changes in serum NfL. The highest estimated NfL rate was observed in the *SNCA*-PD group, and the lowest estimated NfL rate was observed in the *LRRK2*-PD group, which was in accordance with our hypothesis. However, the estimated NfL rate in the *GBA*-PD fell short of our expectations for it was not higher than that in iPD as expected. This finding was inconsistent with a previous study but consistent with data from PPMI^9,13^. We hypothesized that the higher MoCA score in the *SNCA*-PD group and lower MoCA score in the *LRRK2*-PD group might be explained by a faster rise in serum NfL in *SNCA*-PD but a slower increase in *LRRK2*-PD.

Most importantly, we found that longitudinal changes in serum NfL played a mediating role in how different mutations were associated with cognitive progression in PD. We found that the relationship between *GBA* mutation and dementia was not mediated by baseline or longitudinal NfL, and *GBA* yielded no positive effect on dementia. This finding contradicted previous studies that used Mini-Mental State Examination (MMSE) to define cognitive impairment, arguing that *GBA*-PD experienced faster cognitive decline^18,19^. Considering that motor and cognitive impairments vary greatly from *GBA* wildtype to *GBA* severe^20^, we attributed this disparity to our population’s large proportion of mild phenotypes of GBA genotypes. We also discovered that *SNCA* mutation only positively affected dementia, and no mediation effect of baseline or longitudinal NfL on dementia was observed in *SNCA*-PD. *LRRK2* mutation yielded a direct and indirect negative effect on dementia when considering the longitudinal change of serum NfL as the mediator, suggesting that mild cognitive decline in *LRRK2* mutation could be partly mediated by a slower longitudinal change in NfL, which was in line with our hypothesis. Consistently, slow progression in *LRRK2*-PD has been reported by a previous study^21^. Moreover, our mediation model indicated that a higher longitudinal change in NfL levels was associated with an increased risk of conversion to dementia. This finding supports the utility of dynamic NfL measurements as a predictor of cognitive progression in PD^8^. However, in our large PPMI cohort comprising iPD, *GBA*-PD, *LRRK2*-PD, and *SNCA*-PD, we did not observe a significant relationship between baseline NfL and dementia. This result contradicts prior studies which advocated the predictive value of baseline serum NfL levels in determining cognitive decline^5,6,9^.

The mechanisms underlying the association between mutations, NfL, and dementia remain unclear. One hypothesis suggests that *GBA* mutation is responsible for β-glucocerebrosidase dysfunction, causing the accumulation of glucosylceramide^20,22^, which can be harmful to neurons^23^ and might gradually drive PD to develop dementia. In addition, excessive glucosylceramide may increase the formation of α-synuclein, which, in turn, aggravates the accumulation of glucosylceramide^22^. Besides, the *LRRK2* mutation increases β-glucocerebrosidase activity, while the *SNCA* mutation exacerbates cortical α-synuclein deposition, leading to elevated glucosylceramide levels in CSF^14,24^. These mutations may influence glucosylceramide accumulation, thereby affecting neural damage and cognitive impairment, which could be reflected in the levels of NfL^7^. In our mediation model, *LRRK2* mutation exerted an indirect effect on dementia through the longitudinal change of serum NfL, suggesting that NfL might play additional roles beyond being solely a biomarker of neural damage in the context of *LRRK2*-related dementia. Indeed, future research is needed to explore the underlying pathology of mutation in dementia through NfL.

Our analysis has several limitations that should be acknowledged. Firstly, there were many missing values at various follow-up time points, potentially impacting the analysis and conclusions drawn from the study. Despite the missing data, to our knowledge, this study remains the largest reported cohort of different mutations with serum NfL and dementia. Secondly, the number of subjects in the *SNCA*-PD group was limited to only 18, which may reduce the credibility of statistical results for this group. Thirdly, using an R package to establish cut-off values in NfL might have limitations. Lastly, we did not investigate the exact genotype of these genes. Further research is required to explore the relationship between NfL and the genotype of these genes.

The value of this study lies in validating the work of previous studies and uncovering the potential relationship between mutations, NfL, and dementia.

In conclusion, the findings of our study suggest that longitudinal changes in serum NfL levels differ among different PD mutations, with the highest levels observed in *SNCA*-PD and the lowest levels observed in *LRRK2*-PD. These dynamic changes in NfL could be equally effective in predicting cognitive decline as a single time point measurement of serum NfL. Furthermore, we observed that the *LRRK2*-PD group experienced slower progression of dementia, which could be partly mediated by a slower longitudinal change in NfL levels. These findings provide insights into the potential utility of dynamic NfL in predicting cognitive decline in PD and the underlying mechanisms of how specific mutations might influence disease progression.

## Methods

### Study design

Data were obtained from the Parkinson’s Progression Markers Initiative (PPMI) database (http://www.ppmi-info.org). PPMI is an ongoing, observational, international, multicenter study aimed at identifying blood, cerebrospinal fluid, genetic, and imaging biomarkers for the progression of PD. Specific information on the objective, study design, and methodology of the study can be found in previous studies^25^. This study was registered in ClinicalTrials.gov (NCT01141023). Each PPMI site received approval from an ethics committee on human experimentation prior to study initiation, and the participants provided written informed consent.

### Participants

The data were downloaded from the PPMI database on 2 March 2023. The study initially included 881 cases that underwent an 8-year follow-up, including patients with iPD (*n* = 397), *GBA*-PD (*n* = 96), *LRRK2*-PD (*n* = 165), and *SNCA*-PD (*n* = 28), and HC (*n* = 195). We excluded 78 individuals, including patients with iPD (*n* = 16), *GBA*-PD (*n* = 20), *LRRK2*-PD (*n* = 23), and *SNCA*-PD (*n* = 10), and HC (*n* = 9) from the analysis due to the unavailability of serum NfL or MoCA data at any visit point. To eliminate the possible effect of mutations on the results, we then excluded 2 HC with *GBA* and *LRRK2* mutations. We also stratified the cohort into two subgroups based on cognitive status at baseline for further analysis. The detailed features of the subgroups can be found in Supplementary Table 1 and Supplementary Table 2.

### Analysis of serum NfL

Serum NfL concentrations were measured at baseline, 0.5-, 1-, 2-, 3-, and 5-year intervals using the Simoa Human NF-light Advantage kit with the Single Molecule Array in a fully automated SIMOA® HD-1 analyzer (Quanterix, Lexington, MA, USA). The details of the samples are available in the PPMI biologic manual (http://www.ppmi-info.org).

### Clinical assessment

All participants in our study received the PPMI standard test battery for assessment. Disease duration was defined as the duration from the onset of symptoms to enrollment. H&Y stage and MDS-UPDRS were assessed at baseline to evaluate movement disorders. PD global cognitive function was measured with MoCA at baseline and 1-, 2-, 3-, 4-, 5-, 6-, 7-, and 8-year intervals, and the time of dementia was recorded in our study. According to the criteria of the MDS level I guideline, conversion to PD dementia was defined as MoCA <22^26^. It is well-established that MoCA has high sensitivity and specificity in distinguishing MCI in PD^27^. In this study, we used MoCA to determine the presence of dementia among participants. MoCA is widely recognized as an effective and reliable tool for assessing cognitive function and has been shown to have comparable merits to other cognitive assessment scales, such as the PD-focused Scales for Outcomes in PD-Cognitive (SCOPA-COG) and the MMSE^8^.

### Statistical analysis

Continuous variables were compared with the Kruskal–Wallis test, followed by post hoc analysis after the Bonferroni correction or the U test. Categorical variables were compared with the chi-square test followed by post hoc analysis. Multiple comparisons of serum NfL at baseline between groups were further tested by ANCOVA adjusted for age, sex, education, and disease duration after Bonferroni correction.

The linear mixed-effect model was used to estimate the longitudinal change of serum NfL per year. The fixed-effect predictors were mutations, years in the study, and their interactions. Mutations were defined as a dummy variable with iPD as the reference. The fixed covariates included age at baseline, sex, and their interactions with years in the study. Random effects included an intercept and linear rate of change across time per subject. We extracted the estimated change of NfL for every participant and compared the rate of change by the mutations.

To investigate the potential pathways linking mutations to dementia, we used a mediation model with serum NfL at baseline or the estimated change of serum NfL as the mediator. Before analysis, we excluded PD with dementia at baseline. Serum NfL was defined as a dichotomous variable based on the R package “survminer,” which determines the optimal cutpoint for continuous variables using the maximally selected rank statistics from the “maxstat” R package^5^. Likewise, mutations were defined as a dummy variable with iPD as the reference. The association between mutations (factor) and serum NfL (mediator) was tested based on logistic regression models, and the association between mutations (factor) or serum NfL (mediator) with conversion to dementia (dependent) was tested based on Cox regression models. Covariates included age, sex, education, and disease duration. During Cox regression, if the mediator was the estimated change of NfL per year, serum NfL at baseline was added to the covariates. We calculated the direct and indirect effects of mediators using the R package “RMediation”^24,28^.

All statistical analyses were performed using SPSS 26 and R version 4.2.1. A *p*-value < 0.05 was statistically significant.

### Reporting summary

Further information on research design is available in the Nature Research Reporting Summary linked to this article.

### Supplementary information

Supplementary Material

Reporting summary

## Data Availability

The data used in this study is available and can be downloaded from the PPMI website (ppmi-info.org).

## Code availability

All analyses were performed using SPSS 26 and R version 4.2.1. R packages include “lme4”, “survminer”, “maxstat”, and “RMediation”.

## References

[1] Aarsland D, Zaccai J, Brayne C (2005). A systematic review of prevalence studies of dementia in Parkinson’s disease. Mov. Disord..

[2] Aarsland D, Kurz MW (2010). The epidemiology of dementia associated with Parkinson’s disease. Brain Pathol..

[3] Hely MA, Reid WGJ, Adena MA, Halliday GA, Morris JGL (2008). The Sydney multicenter study of Parkinson’s disease: the inevitability of dementia at 20 years. Mov. Disord..

[4] Khalil M (2018). Neurofilaments as biomarkers in neurological disorders. Nat. Rev. Neurol..

[5] Kim R, Jeon B (2021). Serum neurofilament light chain predicts future freezing of gait in Parkinson’s disease. Parkinsonism Relat. Disord..

[6] Rodstrom EY, Mattsson-Carlgren N, Janelidze S, Hansson O, Puschmann A (2022). Serum neurofilament light chain as a marker of progression in Parkinson’s disease: long-term observation and implications of clinical subtypes. J. Parkinsons Dis..

[7] Halloway S (2022). Association of neurofilament light with the development and severity of Parkinson disease. Neurology.

[8] Ma L-Z (2021). Serum neurofilament dynamics predicts cognitive progression in de novo Parkinson’s disease. J. Parkinsons Dis..

[9] Aamodt WW (2021). Neurofilament light chain as a biomarker for cognitive decline in Parkinson disease. Mov. Disord..

[10] Gan-Or Z (2015). Differential effects of severe vs mild GBA mutations on Parkinson disease. Neurology.

[11] Ross OA (2011). Association of LRRK2 exonic variants with susceptibility to Parkinson’s disease: a case-control study. Lancet Neurol..

[12] Mirelman A (2015). Nonmotor symptoms in healthy Ashkenazi Jewish carriers of the G2019S mutation in the LRRK2 gene. Mov. Disord..

[13] Thaler A (2018). Survival rates among Parkinson’s disease patients who carry mutations in the LRRK2 and GBA genes. Mov. Disord..

[14] Koros C (2018). Selective cognitive impairment and hyposmia in p.A53T SNCA PD vs typical PD. Neurology.

[15] Huang, Y., Huang, C., Zhang, Q., Shen, T. & Sun, J. Serum NFL discriminates Parkinson disease from essential tremor and reflect motor and cognition severity. *BMC Neurol.* **22**, 39 (2022). 10.1186/s12883-022-02558-9 PMC8793176 35086487

[16] Planas-Ballve, A. & Vilas, D. Cognitive impairment in genetic Parkinson’s disease. *Parkinsons Dis.* **2021**, 8610285 (2021). 10.1155/2021/8610285 PMC8739522 35003622

[17] Baek, M. S., Lee, M. J., Kim, H.-K. & Lyoo, C. H. Temporal trajectory of biofluid markers in Parkinson’s disease. *Sci. Rep.* **11**, 14820 (2021). 10.1038/s41598-021-94345-8 PMC8292456 34285331

[18] Salamon A, Zadori D, Szpisjak L, Klivenyi P, Vecsei L (2022). The genetic background of Parkinson’s disease and novel therapeutic targets. Expert Opin. Ther. Targets.

[19] Oftedal L (2023). Association of CSF glucocerebrosidase activity with the risk of incident dementia in patients with Parkinson disease. Neurology.

[20] Lerche S (2021). The mutation matters: CSF profiles of GCase, sphingolipids, alpha-synuclein in PDGBA. Mov. Disord..

[21] Saunders-Pullman R, Mirelman A, Alcalay RN (2018). LRRK2 Ashkenazi Jewish Consortium. Progression in the LRRK2-associated Parkinson disease population. JAMA Neurol..

[22] Huh, Y. E. et al. Glucosylceramide in cerebrospinal fluid of patients with GBA-associated and idiopathic Parkinson’s disease enrolled in PPMI. *NPJ Parkinsons Dis.* **7**, 102 (2021). 10.1038/s41531-021-00241-3 PMC8608962 34811369

[23] Korkotian E (1999). Elevation of intracellular glucosylceramide levels results in an increase in endoplasmic reticulum density and in functional calcium stores in cultured neurons. J. Biol. Chem..

[24] Sosero, Y. L. et al. LRRK2 p.M1646T is associated with glucocerebrosidase activity and with Parkinson’s disease. *Neurobiol. Aging* **103**, 142.e1–142.e5 (2021). 10.1016/j.neurobiolaging.2021.02.018 PMC8178224 33781610

[25] Marek K (2011). The Parkinson progression marker initiative (PPMI). Prog. Neurobiol..

[26] Litvan I (2012). Diagnostic criteria for mild cognitive impairment in Parkinson’s disease: Movement Disorder Society Task Force guidelines. Mov. Disord..

[27] Hendershott TR, Zhu D, Llanes S, Poston KL (2017). Domain-specific accuracy of the Montreal Cognitive Assessment subsections in Parkinson’s disease. Parkinsonism Relat. Disord..

[28] Mackinnon DP, Cox MC (2012). Commentary on “Mediation analysis and categorical variables: the final frontier” by Dawn Iacobucci. J. Consum. Psychol..

