# Supplementary material for: Neurofilament light chain as a mediator between LRRK2 mutation and dementia in Parkinson’s disease
Source: NPJ Parkinsons Dis. 2023 Sep 12;9:132. doi: 10.1038/s41531-023-00572-3 (PMC10497522; doi:10.1038/s41531-023-00572-3)
Supplement: Supplementary file 1 — Supplementary Material [file 41531_2023_572_MOESM1_ESM.pdf]

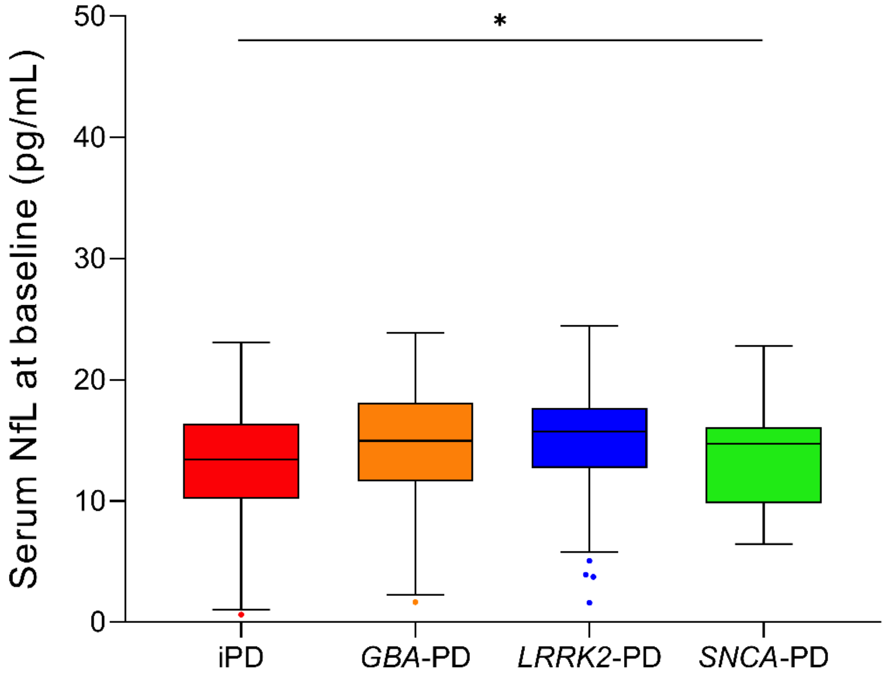

Supplementary Figure 1: Comparisons of serum NfL at baseline between iPD, GBA-PD, LRRK2-PD and SNCA-PD.

Supplementary Table 1: Demographics and Baseline Characteristics between groups in PDD.

Variables	iPD (n = 10)	*GBA*-PD (n = 6)	*LRRK2*-PD (n = 13)	*SNCA*-PD (n = 7)	p among PD groups[A]
Age (years)	66.94 (9.97)	70.46 (3.59)	66.28 (11.91)	52.97 (10.63)	0.096
Sex (male)	8 (80.00%)	3 (50.00%)	5 (38.50%)	4 (57.10%)	0.268
Education (years)	13.60 (2.50)	15.20 (2.28)	12.15 (5.54)	11.17 (5.57)	0.323
Disease duration (Months)	22.10 (15.32)[a]	67.20 (57.27)[a, b]	58.77 (45.77)[b]	48.84 (26.56)[a, b]	0.025
Serum NfL (pg/mL)	17.52 (5.79)	18.92 (4.03)	16.81 (7.18)	19.86 (13.63)	0.835
MoCA (baseline)	19.90 (1.66)	19.60 (2.61)	18.62 (2.33)	18.00 (3.58)	0.197
H&Y stage	1.40 (0.52)[a]	2.20 (0.45)[a, b]	2.00 (0.58)[a, b]	2.17 (0.75)[b]	0.020
MDS-UPDRS III score	16.90 (4.89)	31.00 (18.49)	19.23 (10.64)	39.50 (23.96)	0.334
MDS-UPDRS total score	28.00 (10.00)	46.80 (24.95)	36.54 (15.82)	68.67 (33.80)	0.059

Data are mean (SD) or n (%). Significance level for comparisons is $p < 0.05$. Abbreviations: NfL, Neurofilament light chain; MoCA, Montreal Cognitive Assessment; H&Y, Hoehn and Yahr; MDS-UPDRS, Movement Disorders Society Unified Parkinson's Disease Rating Scale. [A] Indicated continuous variables were compared using Kruskal-Wallis test, and categorical variables were compared with chi-square test. Multiple comparisons between four PD groups were adjusted by Bonferroni correction. Values with same letters are not statistically different ($p > 0.008$).

Supplementary Table 2: Demographics and Baseline Characteristics between groups in PD without dementia.

Variables	iPD (n = 340)	*GBA*-PD (n = 66)	*LRRK2*-PD (n = 115)	*SNCA*-PD (n = 11)	p among PD groups[A]
Age (years)	61.90 (9.61)[a]	60.59 (10.92)[a]	63.39 (9.12)[a]	45.46 (7.92)[b]	< 0.001
Sex (male)	241 (65.00%)[a]	40 (57.10%)[a, b]	61 (47.30%)[b]	5 (45.50%)[a, b]	0.003
Education (years)	15.60 (2.95)[a]	16.17 (3.70)[a]	15.53 (4.37)[a]	12.00 (3.61)[b]	0.001
Disease duration (Months)	24.46 (24.95)[a]	46.52 (33.08)[b]	62.76 (68.07)[b]	45.27 (26.95)[b]	< 0.001
Serum NfL (pg/mL)	13.02 (7.22)	14.25 (10.63)	14.70 (8.55)	9.98 (5.16)	0.059
MoCA (baseline)	27.26 (2.05)	27.00 (1.97)	26.94 (2.03)	28.09 (2.43)	0.013
H&Y stage	1.56 (0.50)[a]	1.76 (0.53)[b]	1.78 (0.59)[b]	1.45 (0.52)[a, b]	< 0.001
MDS-UPDRS III score	21.00 (9.02)[a, b]	24.59 (11.33)[a]	19.80 (9.70)[a]	13.73 (8.78)[b]	0.002
MDS-UPDRS total score	32.35 (13.44)[a]	40.58 (15.87)[b]	34.73 (15.93)[a, b]	27.27 (12.54)[a, b]	< 0.001

Data are mean (SD) or n (%). Significance level for comparisons is $p < 0.05$. Abbreviations: NfL, Neurofilament light chain; MoCA, Montreal Cognitive Assessment; H&Y, Hoehn and Yahr; MDS-UPDRS, Movement Disorders Society Unified Parkinson's Disease Rating Scale. [A] Indicated continuous variables were compared using Kruskal-Wallis test, and categorical variables were compared with chi-square test. Multiple comparisons between four PD groups were adjusted by Bonferroni correction. Values with same letters are not statistically different ($p > 0.008$).
